# Engineering Extracellular Vesicles with the Tools of Enzyme Prodrug Therapy

**DOI:** 10.1002/adma.201706616

**Published:** 2018-02-23

**Authors:** Gregor Fuhrmann, Rona Chandrawati, Paresh A. Parmar, Timothy J. Keane, Stephanie A. Maynard, Sergio Bertazzo, Molly M. Stevens

**Affiliations:** Department of Materials, Department of Bioengineering, and Institute of Biomedical Engineering, Imperial College London, London SW7 2AZ, UK; Helmholtz Institute for Pharmaceutical Research Saarland, Helmholtz Centre for Infection Research, Biogenic Nanotherapeutics Group, Campus E8.1, 66123 Saarbrücken, Germany; Department of Materials, Department of Bioengineering, and Institute of Biomedical Engineering, Imperial College London, London SW7 2AZ, UK; Department of Medical Physics and Biomedical Engineering, University College London, Malet Place Engineering Building, London WC1E 6BT, UK; Department of Materials, Department of Bioengineering, and Institute of Biomedical Engineering, Imperial College London, London SW7 2AZ, UK

**Keywords:** *β*-glucuronidase, enzyme prodrug therapy, exosomes, hydrogels, microvesicles

## Abstract

Extracellular vesicles (EVs) have recently gained significant attention as important mediators of intercellular communication, potential drug carriers, and disease biomarkers. These natural cell-derived nanoparticles are postulated to be biocompatible, stable under physiological conditions, and to show reduced immunogenicity as compared to other synthetic nanoparticles. Although initial clinical trials are ongoing, the use of EVs for therapeutic applications may be limited due to undesired off-target activity and potential “dilution effects” upon systemic administration which may affect their ability to reach their target tissues. To fully exploit their therapeutic potential, EVs are embedded into implantable biomaterials designed to achieve local delivery of therapeutics taking advantage of enzyme prodrug therapy (EPT). In this first application of EVs for an EPT approach, EVs are used as smart carriers for stabilizing enzymes in a hydrogel for local controlled conversion of benign prodrugs to active antiinflammatory compounds. It is shown that the natural EVs’ antiinflammatory potential is comparable or superior to synthetic carriers, in particular upon repeated long-term incubations and in different macrophage models of inflammation. Moreover, density-dependent color scanning electron microscopy imaging of EVs in a hydrogel is presented herein, an impactful tool for further understanding EVs in biological settings.

Extracellular vesicles (EVs) have recently gained significant attention as important mediators of intercellular communication, potential drug carriers for various dispositions, such as cancer, inflammation, or tissue injury, and biomarkers for sensing different pathophysiological conditions.[1] These naturally released nanoparticles from cells—exosomes and small microvesicles[2]—are postulated to be biocompatible, stable under physiological conditions, able to cross biological barriers, and thought to have the potential for reduced immunogenicity as compared to other nanoparticles including synthetic liposomes or polymeric nanoparticles.[3] Compared to synthetic drug carriers, such as liposomes, EVs do not require postfabrication modifications to obtain targeting abilities.[4] In recent years, substantial effort has been undertaken to bring EVs forward to clinical assessments, including large-scale production, isolation, and characterization of clinical-grade EVs.[5] Although initial clinical trials are ongoing, the use of EVs for therapeutic applications may be limited due to undesired off-target activity and potential “dilution effects” upon systemic administration which may affect their ability to reach their target tissues.[6] To fully exploit the therapeutic potential of EVs, we hypothesize that they can be embedded into implantable biomaterials designed to achieve local delivery of therapeutics. The delivery of a drug of interest in a specific and controlled manner is a key step toward increased therapeutic benefits and decreased off-target effects. Enzyme prodrug therapy (EPT) is a suit of highly successful techniques whereby localized conversion of benign prodrugs to active drugs is accomplished by enzymes.[7–10] By doing so, it is possible to synthesize and release drugs at the desired sites by using systemically administered inactive, nontoxic prodrugs. Recent reports show that incorporation of enzymes into polymeric hydrogel carriers and films renders local production of a range of classes of therapeutics, including antiinflammatory drugs,[8] antiproliferative drugs,[7,8,11] and signaling molecules.[9] This concept of enzyme carrier has not been studied comparing natural EVs with their synthetic liposomal counterparts.

To harness EV’s inherent properties and to combine them with site-specific drug delivery functions, we show here for the first time the encapsulation of EVs into a hydrogel matrix and we present the first application of EVs for an EPT approach. In particular, we expand the concept of EPT by using EVs as smart carriers for the stabilization of enzymatic cargo in a hydrogel for local sustained release of antiinflammatory drugs (Figure 1a). We load EVs with *β*-glucuronidase, an enzyme with historically validated performance in EPT,[8,12,13] and incorporate them into poly(vinyl alcohol) (PVA) hydrogels to achieve site-specific release of an antiinflammatory drug (curcumin) from its glucuronide precursor. We show the very first density-dependent color scanning electron microscopy (DDC-SEM) imaging of EVs in a hydrogel. We compare the mechanical and biomedical properties of enzyme-loaded EVs with liposomes in hydrogels and show that the natural vesicles’ antiinflammatory potential is comparable or superior to the synthetic carriers, in particular upon repeated long-term incubations. With our study we substantially advance the concept of EPT for long-term applications by using smart nanoparticles as protective envelopes for enzymes, a concept that may in the future be applied for other enzyme prodrug systems. Moreover, these results indicate that local application of EVs is a promising strategy to overcome recent obstacles in their therapeutic development.

We first investigated enzyme encapsulation into EVs and their catalytic activity. EVs from human mesenchymal stem cells (hMSCs) were isolated and loaded with *β*-glucuronidase using our newly developed saponin treatment method (Figure S1a, Supporting Information).[14] This method allowed for a mild and efficient encapsulation of *β*-glucuronidase without compromising the structural integrity of the vesicles. As a comparison, we formed a synthetic analog, namely, *β*-glucuronidase-loaded liposomes composed of 1,2-dimyristoyl-*sn*-glycero-3-phosphocholine (DMPC) and 1,2-dipalmitoyl-*sn*-glycero-3-phosphocholine (DPPC) (DMPC:DPPC = 2:3) (Figure S1b, Supporting Information). We selected liposomes as a well-established class of phospholipid-based drug carriers that have proven clinical efficiency in various disease models and are thus an ideal comparison for EVs. Encapsulation efficiency of EVs and liposomes and the functional activity of *β*-glucuronidase within the vesicles were monitored through conversion of a model fluorogenic substrate, fluorescein di-*β*-d-glucuronide (FDGlcU; Figure S1c, Supporting Information), to fluorescein. Figure S1d (Supporting Information) shows comparable encapsulation efficiency with a nearly same level of enzyme activity for EVs and liposomes, the key parameter when comparing bioactivity of natural versus synthetic carrier. It also demonstrates the suitability of EVs as enzyme carriers and that the saponin treatment does not compromise the catalytic activity of *β*-glucuronidase.

Next, we assembled the biocatalytic materials by incorporating enzyme-loaded vesicles into PVA hydrogels. PVA hydrogels are biocompatible and used for biomedical avenues; they can be stabilized without chemical modification, thus are advantageous for protein and liposome incorporation without compromising their biological constitution.[15] *β*-Glucuronidase-loaded vesicles (EVs or liposomes at concentrations of 1.7 × 10^8^ per gel or 3.5 × 10^8^ per gel), or free (nonencapsulated) *β*-glucuronidase (0.1 mg mL^−1^), were mixed into a PVA solution, followed by stabilization with poly(ethylene glycol) (PEG),[16] rendering biocompatible, crosslinked hydrogels of 8 mm diameter and 2 mm thickness (Figure 1b). Mechanical testing indicated that vesicle or enzyme incorporation did not alter the compressive properties of hydrogels (Figure 1c) and thus substrate diffusion is expected to be comparable for all gel formulations. When the gels were incubated in phosphate-buffered saline (PBS) over time at 24 or 37 °C, we observed a ≈20% weight loss in the first day, and the gels remained stable under physiological conditions over 15 d (Figure S2a, Supporting Information). We collected supernatants over time to investigate the presence of enzymes that may be released from the hydrogels. We found a significant reduction in enzyme release when *β*-glucuronidase was encapsulated into EVs or liposomes as compared to when the enzyme was not encapsulated in the vesicles and was directly mixed with the polymer solution (Figure S2b, Supporting Information). These characterizations prove that our vesicle-containing PVA gels are stable and they retain bioactive enzymes for longer incubation periods (at least over 7 d).

To visualize the vesicles within the gels, we fluorescently labeled EVs using a PKH67 membrane dye. Using confocal microscopy, we observed a uniform distribution of fluorescent signals, both in gels with higher and lower EV concentration (Figure 1d; 3.5 × 10^8^ per gel and 1.7 × 10^8^ per gel). Nevertheless, fluorescent spot sizes varied which could suggest the formation of EV-rich domains within the hydrogel. To further verify this observation we performed DDC-SEM[17] of carefully dehydrated hydrogels. This relatively new technique allowed visualization of EVs within hydrogels after labeling with high-density entities such as uranylacetate (EV-uranyl) or hemin (EV-heme) as they both comprise metal ions (uranium and iron, respectively). Using in-lens secondary electron detection combined with density-sensitive backscattered imaging we were able to perform the first-time visualization of EV-uranyl in a dehydrated hydrogel (Figure 1e,g,i; Figure S3, Supporting Information) using a simple and accessible method. Our images confirmed uniform EV distribution with distinct and localized vesicular structures of high density (arrows in Figure 1g). Similar dense, particulate structures were observed for imaging of EV-heme containing gels (Figure S4d, Supporting Information). Control nonloaded hydrogels showed unspecific sample charging (scattered signal in Figure 1h) without clear vesicular structures detected (Figure 1j; Figure S3d, Supporting Information). Additional SEM images were recorded to verify that sample preparation did not impair the hydrogel structure (Figure S3, Supporting Information), in agreement with literature.[18] Both EV-loaded and nonloaded hydrogels exhibited comparable morphology (Figure S4a,b, Supporting Information). To the best of our knowledge, this is the first example of density-dependent SEM imaging of EV-containing hydrogels targeted for therapeutic applications. This novel technique enables a straightforward evaluation of general hydrogel structure and morphology, and a detailed analysis of spatial EV location and distribution in 3D. Taken together, we confirm that incorporation of lipid-based vesicles does not alter the mechanical and structural properties of hydrogels, rendering them promising tools for biomedical applications.

Next, we assessed the enzymatic activity of EV- and liposome-encapsulated *β*-glucuronidase within PVA hydrogels through monitoring the conversion of FDGlcU to fluorescein in PBS over 7 d at 37 °C. In the presence of FDGlcU, a constant increase of fluorescein release during the measurement period was observed for all gels (Figure 2a), with increasing activity for 3.5 × 10^8^ compared to 1.7 × 10^8^ vesicles per gel. Liposome-loaded gels (3.5 × 10^8^ per gel) resulted in the highest fluorescence signal (100% normalized, maximum signal for all timepoints measured), followed by EV-loaded gels (3.5 × 10^8^ per gel, 66%), both normalized to gels with no vesicles (control gels). These results indicate that lipid-vesicle encapsulated *β*-glucuronidase incorporated into hydrogels remains active during 7 d of incubation at body temperature. To further evaluate the long-term catalytic performance of the gels, we repeated the enzymatic catalysis by removing fluorescein and adding fresh FDGlcU to the same PVA hydrogels and monitored the fluorescein signal over another 7 d at 37 °C. The catalytic activity of the liposome-hydrogel (3.5 × 10^8^ per gel) remained nearly constant with maximum activity of 96%, and the catalytic activity of the EV-loaded gels (3.5 × 10^8^ per gel) was 29% lower (Figure 2b; Figure S5, Supporting Information). In contrast, when free *β*-glucuronidase was incorporated directly into PVA gels, up to 78% of enzymatic activity was lost upon gel recycling (Figure S6, Supporting Information). Our results strongly recommend that encapsulation of *β*-glucuronidase into vesicles substantially preserved enzyme activity during 14 d of incubation even after substrate recycling conditions.

Subsequently, we evaluated the therapeutic utility of our hydrogels in a cell model of inflammation. Mouse macrophage cells (RAW 264.7) were challenged with bacterial lipopolysaccharide toward inflammatory conditions and incubated with hydrogels containing EV- or liposome-encapsulated enzyme (EV-glucuronidase or lipo-glucuronidase, respectively) or free glucuronidase, or gels with no vesicles. Upon addition of curcumin-*β*-d-glucuronide substrate the effect of released curcumin on cell viability was assessed over 48 h as a marker for therapeutic efficiency of our gels, a standard method to assess curcumin’s inflammation modulation both in vitro and in vivo.[8,19] We observed a significant or a near-significant antiinflammatory response for EV- and lipo-glucuronidase, and free glucuronidase gels compared to control gels (Figure 3a). Indeed, substrate cleavage by vesicle-encapsulated glucuronidase was visibly indicated by a color shift from orange (curcumin-*β*-d-glucuronide) to yellow (curcumin) (inset in Figure 3a). Moreover, a dose-dependent trend was observed when EV-glucuronidase or free glucuronidase containing gels were incubated with an increasing concentration of curcumin substrate (Figure 3c). In order to assess gene expression and protein concentration of tumor necrosis factor (TNF) alpha as a major marker for curcumin-induced reduction of inflammation,[20] we selected primary bone-marrow-derived murine macrophages (BMDM) as these are more representative for normal physiology as compared to RAW cells[21] (Figure S7, Supporting Information). When incubated with BMDMs for 24 h, hydrogels containing free glucuronidase or glucuronidase encapsulated into EVs or liposomes induced a significant reduction in TNF alpha gene expression, with the strongest effect seen for EV-hydrogels (Figure S7a, Supporting Information). Of note, EV-hydrogels also reduced inflammation even in the absence of the substrate. Indeed, EVs from hMSC cells have been reported in literature to show an inherent antiinflammatory activity.[22] We also observed on average lower TNF alpha concentrations for EV-hydrogel-treated BMDMs although this was not significant possibly due to the 24 h timepoint chosen (Figure S7b, Supporting Information). As such, these results clearly provide evidence for a biomedically functional hydrogel that allows therapeutic titration of active drugs depending on the desired setting (high or low drug release). To assess the ability of gels to convey prolonged enzyme, and thus therapeutic activity, they were stored for 7 d at 37 °C, incubated with murine RAW macrophages, and mixed with fresh curcumin substrate. When analyzing the cellular responses upon gel recycling, we observed a reduction of cell viability for EV- and lipo-glucuronidase, while hydrogels containing free glucuronidase exhibited a complete loss of their enzymatic activity (Figure 3b). These results demonstrate that our vesicle-containing hydrogels elicit superior stability and cellular activity when mimicking longterm incubations at physiological conditions compared to free glucuronidase-containing hydrogels. Finally, none of our hydrogels had a significant impact on cell survival when no substrate was present (Figure 3d), indicating their biocompatibility. By using RAW cells with different passage number we increased biological variability but could also show that our EPT hydrogel works robustly over a broad range of in vitro settings.

In conclusion, we have developed the first EV-based hydrogels for localized and controlled delivery of antiinflammatory drugs. Hydrogels are well studied for biomedical applications and they offer several advantages such as controlled drug release or mimicking of biomechanical functions.[23] Here, encapsulation of pharmaceutically relevant enzymes into EVs allowed their unprecedented protection and prolonged biological activity under physiological-like conditions which is key for biomedical implementation of enzymes.[24] Our proof-of-principle experiments show that encapsulation of EVs into a biocompatible PVA hydrogel is a promising approach not only for EPT but for other therapeutic avenues. It may in the future be applied for cancer treatment or regenerative medicine, as *β*-glucuronidase is capable of catalyzing a broad range of prodrugs with the glucuronic acid protecting trigger.[13] Upcoming evaluations could therefore comprise applications of other prodrugs and more detailed biomedical assessments. For these hydrogels, we have shown that EVs are comparable to or better than synthetic liposomes, which may in the future be beneficial for developing semisynthetic EV-inspired approaches. Moreover, our simple method of EV incorporation into hydrogels may be extended to other biological applications, such as the localized delivery of EVs or the use of EV-containing scaffolds for tissue engineering, which would further amplify EV approaches. We also presented the very first imaging of EVs in hydrogels at the nanoscale using DDC-SEM, a powerful tool for the analysis of hydrogel morphology and spatial EV distribution and localization in 3D. This simple and accessible technique may in the future assist to further characterize native EVs in biologically relevant settings such as 3D cell cultures or tissue scaffolds, and can substantially advance EV applications in biomedical research. Utilization of EVs in hydrogels or other bioactive scaffolds represents a promising opportunity to overcome current obstacles associated with their development as novel therapeutic entities.

## Experimental Section

### Preparation and Characterization of Hydrogels

Detailed information on vesicle preparation, enzyme loading, and hydrogel production can be found in the Supporting Information. Briefly, *β*-glucuronidase was encapsulated into EVs derived from mesenchymal stem cells or liposomes (DMPC:DPPC = 2:3) via a saponin treatment.[14] The vesicles were mixed into a PVA solution (12 wt%), followed by crosslinking with PEG, rendering hydrogels with 1.7 or 3.5 × 10^8^ vesicles per gel.

### Scanning Electron Microscopy (SEM)

For SEM imaging, typically 200–400 μL of EVs were mixed with uranyl acetate (0.05 wt%) or hemin (final 1 mg mL^−1^ w/v) for 10 min at room temperature to allow labeling. EVs were purified by size-exclusion chromatography (SEC), characterized and loaded into hydrogels as described above. Hydrogels containing labeled EVs, native EVs, or PBS control gels were dehydrated with increasing amounts of methanol (10–100% v/v in water), for 1 h at each step and air dried, snap frozen in liquid nitrogen, and cut with a razor blade. Samples were attached to aluminum stubs with carbon tape, silver paint was spread on the sample sides, and coated with 5 nm carbon (Quorum Technologies Turbo-Pumped Thermal Evaporators model K975X). Gels were imaged by SEM (Zeiss Auriga) operated at 5 kV, equipped to record in-lens and secondary electrons, and in backscatter mode. The density-dependent color SEM analysis was executed as described previously,[17] briefly the in-lens or secondary electron image was assigned to the green channel while the backscattering signal was assigned to the red channel; images were stacked using ImageJ.

### Enzymatic Activity of Hydrogels

To assess the enzymatic activity of EV-, liposome-, or free-enzyme-loaded hydrogels, these gels were washed three times with PBS and incubated with fluorescein di-*β*-d-glucuronide at a final concentration of 8 × 10^−3^ m. The fluorescence intensity of products (fluorescein) was monitored at 0, 24, and 48 h, and 5 and 7 d using an EnSpire microplate reader (PerkinElmer) at 495/520 nm (excitation/emission). Afterward, gels were washed thoroughly with PBS and incubated with fresh substrate for another week. All samples were normalized to the corresponding PBS hydrogel (control gel) and setting the highest observed value to 100% released fluorophore (equivalent for both cycles). Each experiment was repeated with *n* = 3–5.

### Antiinflammatory Activity of Hydrogels

Murine RAW264.7 macrophage cells (RAW, passages 16-20, ATCC) were cultured in Dulbecco’s modified Eagle medium (DMEM) supplemented with 10% (v/v) FBS and 1% (w/v) penicillin/streptomycin until near-confluent state. Subsequently, they were washed with PBS, detached by scratching, and counted. RAWs were seeded in 48-well plates (60 000 cells per well) in DMEM (10% (w/v) FBS, P/S) and allowed to adhere overnight at 37 °C and 5% CO_2_. Then, cells were activated by the addition of 1 μg mL^−1^ lipopolysaccharide from *Escherichia coli* 026:B6 (Sigma L2654). Hydrogels containing *β*-glucuronidase encapsulated into EVs or liposomes, free enzyme, or control gels were washed in PBS and sterilized under the UV lamp for 10–15 min and added to the cell supernatants. The substrate curcumin-*β*-d-glucuronide (Apollo Scientific) was added at concentrations between 0 and 80 × 10^−6^ m and plates were incubated for 48 h at 37 °C and 5% CO_2_. For recycling experiments, hydrogels were incubated at 37 °C in PBS for 7 d. Subsequently, they were incubated with freshly seeded RAWs and fresh curcumin substrate. For analysis, gels were removed; cells were washed with PBS and incubated in AlamarBlue (Invitrogen) solution (10% (v/v) in DMEM without phenol red) for 3 h at 37 °C and 5% CO_2_. The relative levels of metabolic activity were assessed by fluorescence readings at 560/600 nm (excitation/emission). The metabolic activity of the cells was used as a measure for cell viability and antiinflammatory activity. Each experiment was repeated with *n* = 5–13.

### Statistical Analysis

All data are displayed as mean ± standard deviation, indicating the number *n* of independent replicates. Analysis of variance (ANOVA) followed by a post hoc test (as indicated in figure legends) was used for pairwise comparisons; differences were considered significant at *p* > 0.05 (Sigma Plot).

## Supplementary Material

Supporting Information is available online from the Wiley Online Library or from the author.

Supporting Information

## Figures and Tables

**Figure 1:**
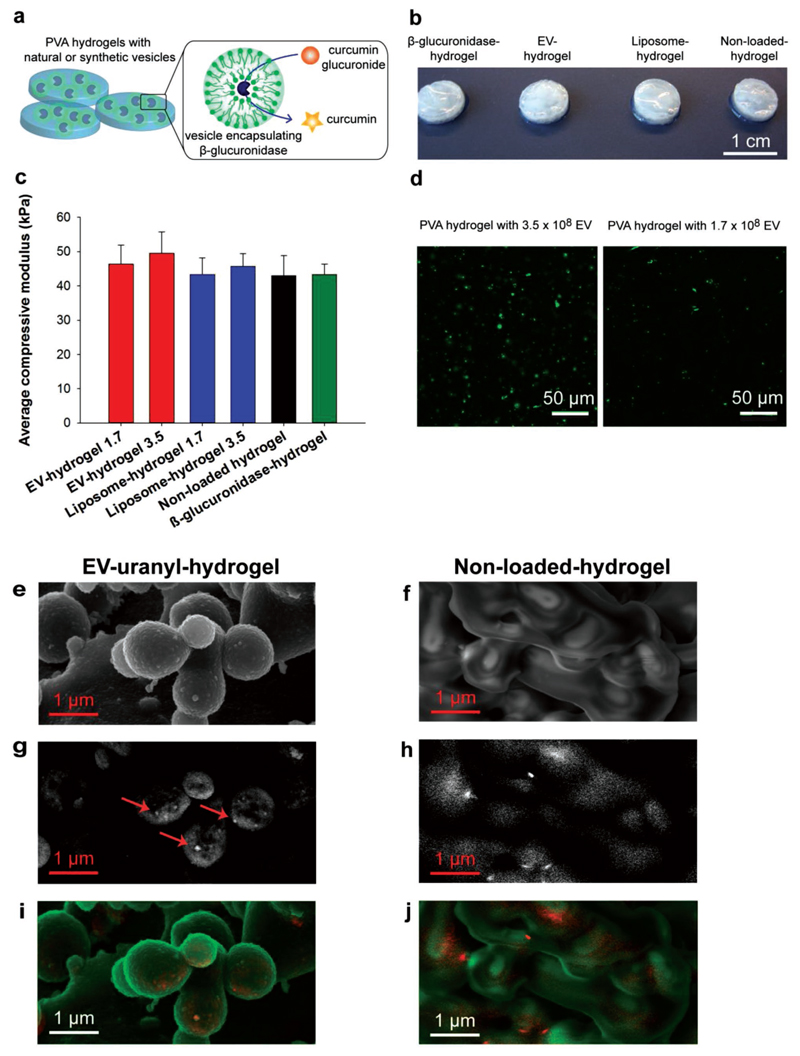
Characterization of poly(vinyl alcohol) hydrogels containing enzyme-loaded vesicles. a) Schematic overview of EV- or liposome-encapsulated enzyme (β-glucuronidase) incorporated into PVA hydrogels. Enzyme-functionalized hydrogels are incubated with a glucuronide prodrug to release an active drug in a selective and controlled manner. b) Photographs of PVA hydrogels containing free (nonencapsulated) *β*-glucuronidase, EV- or liposome-encapsulated *β*-glucuronidase (EV hydrogel or liposome hydrogel, respectively), or nonloaded control gels. c) Mechanical properties of PVA hydrogels. EV hydrogels and liposome hydrogels were loaded with 1.7 × 10^8^ or 3.5 × 10^8^ vesicles per gel (indicated by number in (c)). Unconfined elastic modulus of compression of hydrogels compressed to 10% strain at 0.5% strain min^−1^. Values are represented as mean ± SD, *n* = 5, no differences by one-way ANOVA (*p* < 0.05). d) Confocal micrographs of EVs fluorescently labeled with PKH67 and incorporated into PVA hydrogels at higher and lower concentration (3.5 × 10^8^ and 1.7 × 10^8^ EVs per gel, respectively). e–j) Scanning electron microscopy imaging of PVA hydrogels containing optically more dense uranyl-labeled EVs (EV-uranyl, 3.5 × 10^8^ EVs per gel, indicated by arrows) or no vesicles (nonloaded control gels). EV-uranyl-hydrogel samples exhibit distinct and localized vesicular structures (arrows) while control nonloaded hydrogels show unspecific sample charging without clear vesicular structures. Images were obtained by e,f) in-lens electron detector and g,h) in backscattered electron mode; and by i,j) density-dependent SEM analysis with the in-lens or secondary electron image assigned to the green channel and the backscattering signal assigned to the red channel.

**Figure 2:**
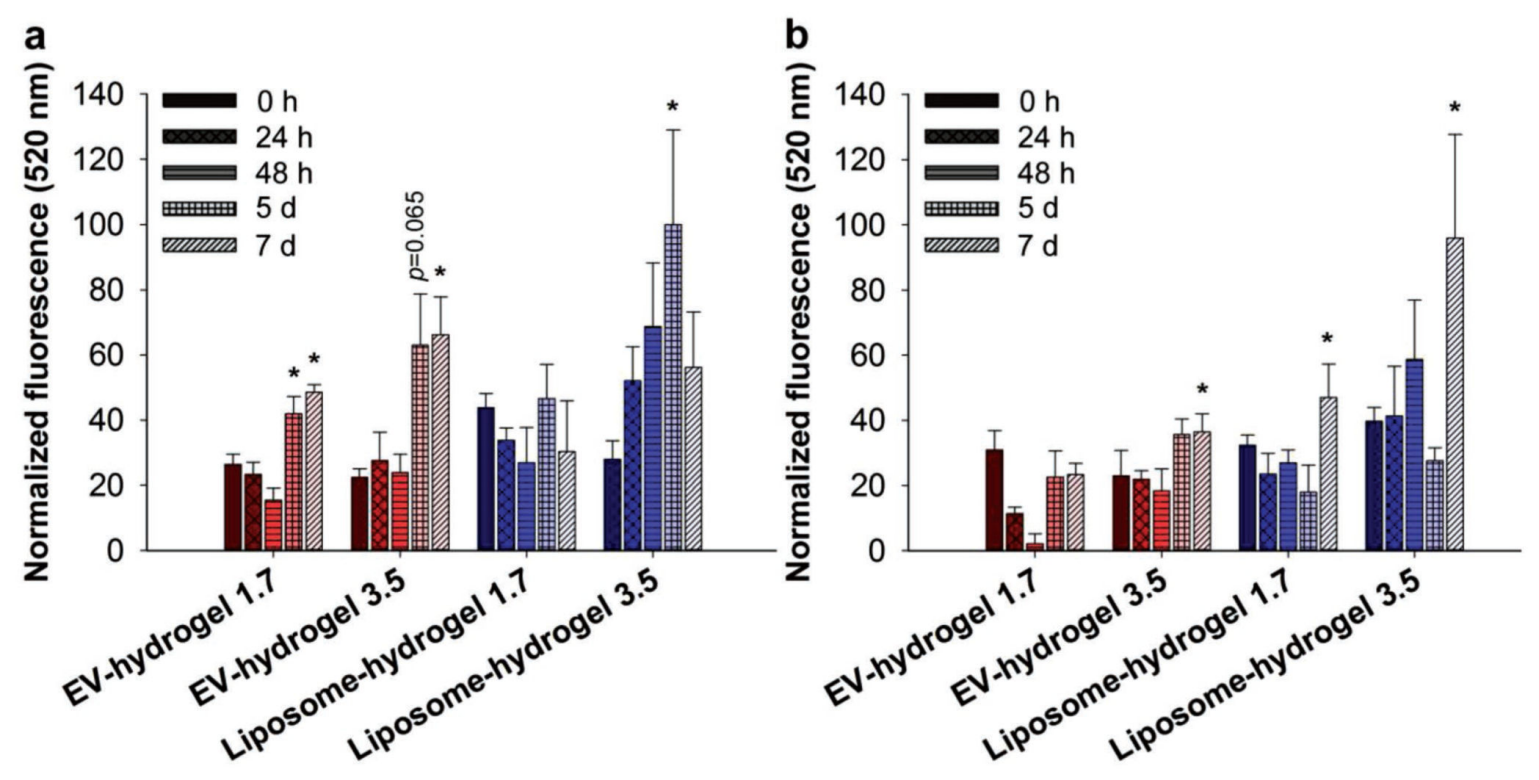
Enzymatic activity of vesicle-loaded hydrogels. a) Incubation of EV- or liposome-encapsulated *β*-glucuronidase in hydrogels with fluorescein di-β-d-glucuronide for up to 7 d. EV hydrogels and liposome hydrogels were loaded with 1.7 × 10^8^ or 3.5 × 10^8^ *β*-glucuronidase-encapsulated vesicles per gel. Enzymatic cleavage was assessed by measuring increasing cumulative fluorescence produced by fluorescein. b) After 7 d, gels were washed thoroughly with PBS and incubated with fresh fluorescein di-β-d-glucuronide substrate to assess the enzyme activity upon long-term application. Values are represented as mean ± SD, *n* = 3–5, *p < 0.05 versus hydrogels at 0 h (ANOVA on ranks with Dunn’s post hoc test was performed on raw data). Normalization was executed against PBS control sample (set to 0%, not included) and the highest observed fluorescein release (100%). In (a) and (b), 0% and 100% are equal to facilitate comparison between both cycles.

**Figure 3:**
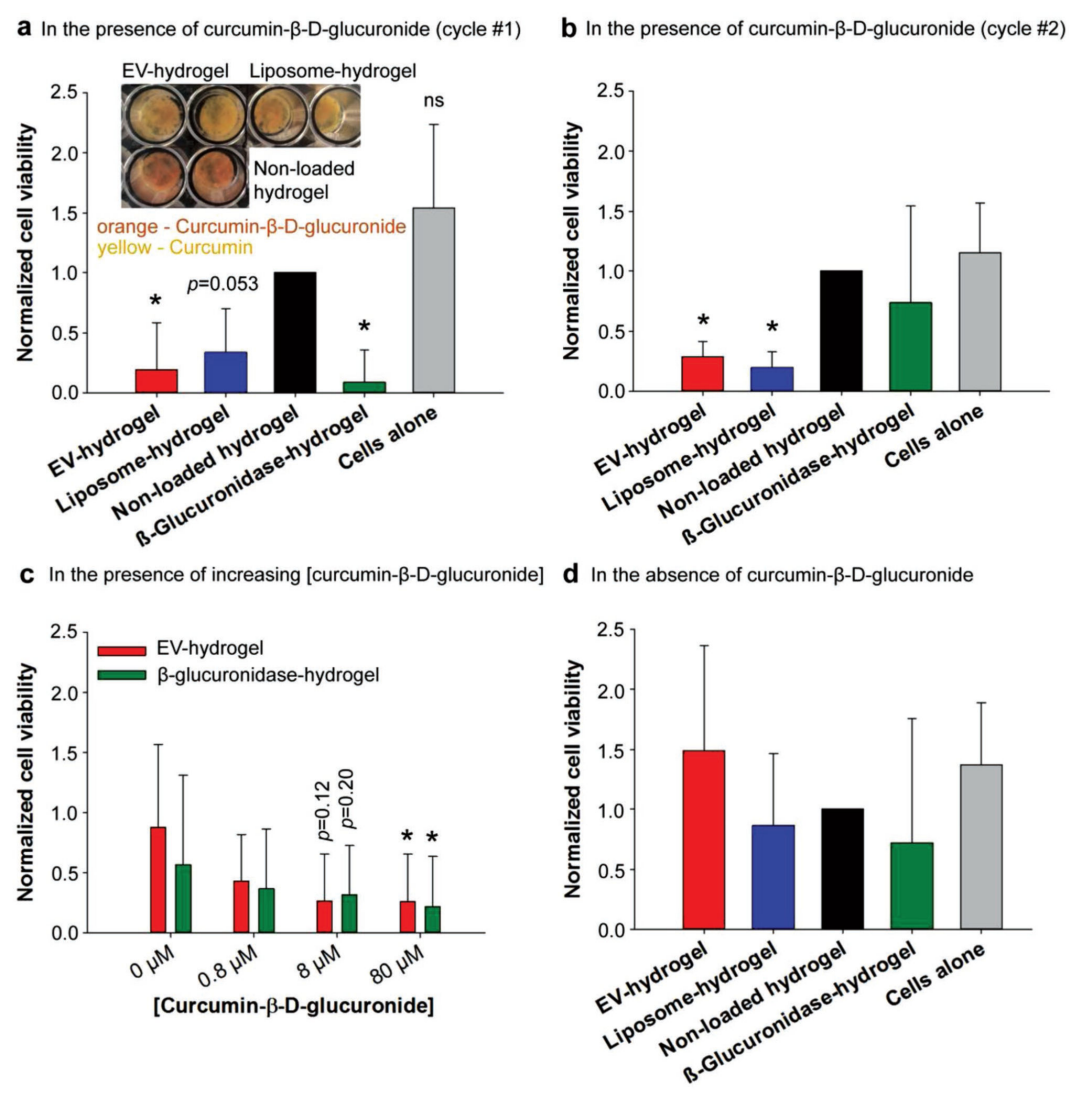
In vitro activity of hydrogels in a cellular model of inflammation. a–c) Mouse macrophages (RAW 264.7) were stimulated with bacterial lipopolysaccharide and incubated with hydrogels containing EV- or liposome-encapsulated *β*-glucuronidase (EV hydrogel and liposome hydrogel, both loaded with 3.5 × 10^8^ *β*-glucuronidase-encapsulated vesicles per gel), control gels (nonloaded hydrogel), or free *β*-glucuronidase-containing hydrogels (β-glucuronidase hydrogel). Upon addition of curcumin-β-d-glucuronide, the release of antiinflammatory curcumin was assessed as a function of cell viability. a) Antiinflammatory response upon a single incubation and c) with increasing amounts of prodrug (0, 0.8, 8, and 80 × 10^−6^ m curcumin-β-d-glucuronide). b) Cell viability upon washing of gels from (a) and repeated incubation with freshly seeded cells and prodrug substrate. d) Control experiments hydrogels containing EV- or liposome-encapsulated *β*-glucuronidase (EV hydrogel and liposome hydrogel, both loaded with 3.5 × 10^8^ *β*-glucuronidase-encapsulated vesicles per gel), control gels (nonloaded hydrogel), or free *β*-glucuronidase-containing hydrogels (β-glucuronidase hydrogel) without addition of prodrug substrate. Values are represented as mean ± SD, *n* = 5–13; *p < 0.05 versus nonloaded hydrogel (ANOVA with Dunnett post hoc for (a), (b), and (d)) and *p < 0.05 versus EV hydrogel 0 or *β*-glucuronidase hydrogel 0 (ANOVA with Tukey post hoc for (c)); ns indicates no significant difference between nonloaded hydrogel and cells alone in (a).
