# Supplementary material for: Engineering Extracellular Vesicles with the Tools of Enzyme Prodrug Therapy
Source: Adv Mater. Author manuscript; Available in PMC 2018 Apr 16. (PMC5901706; doi:10.1002/adma.201706616)
Supplement: Supporting Information [file NIHMS76410-supplement-Supporting_Information.pdf]

Copyright WILEY-VCH Verlag GmbH & Co. KGaA, 69469 Weinheim, Germany, 2018.

ADVANCED MATERIALS

Supporting Information

for *Adv. Mater.*, DOI: 10.1002/adma.201706616

Engineering Extracellular Vesicles with the Tools of Enzyme Prodrug Therapy

*Gregor Fuhrmann, Rona Chandrawati, Paresh A. Parmar, Timothy J. Keane, Stephanie A. Maynard, Sergio Bertazzo, and Molly M. Stevens**

Submitted to ADVANCED MATERIALS

Supporting Information

for *Adv. Mater.*, DOI: 10.1002/adma.201706616

Engineering extracellular vesicles with the tools of enzyme prodrug therapy

*Gregor Fuhrmann, Rona Chandrawati, Paresh A. Parmar, Timothy J. Keane, Stephanie A. Maynard, Sergio Bertazzo, Molly M. Stevens**

Dr G. Fuhrmann,[++] Dr R. Chandrawati,[+] [++] Dr P. A. Parmar, Dr T. J. Keane,
S. A. Maynard, Prof M. M. Stevens
Department of Materials, Department of Bioengineering, and Institute of Biomedical Engineering
Imperial College London
London SW7 2AZ, UK
E-mail: m.stevens@imperial.ac.uk

Dr G. Fuhrmann
Helmholtz-Centre for Infection Research, Helmholtz-Institute for Pharmaceutical Research Saarland, Biogenic Nanotherapeutics group, Campus E8.1, 66123 Saarbrücken, Germany

Dr S. Bertazzo
Department of Medical Physics and Biomedical Engineering
University College London, Malet Place Engineering Building
London WC1E 6BT, UK

[+] Present address: School of Chemical and Biomolecular Engineering, The University of Sydney, Sydney, NSW 2006, Australia

[++] G.F. and R.C. contributed equally to this work

Supplementary Methods
Figure S1. Characterization and loading of EVs and liposomes.
Figure S2. Stability of hydrogels upon storage.
Figure S3. Supplementary density-dependent scanning electron microscopy imaging.
Figure S4. Complementary scanning electron micrographs of control gels.
Figure S5. Activity of EV and liposome loaded hydrogels.
Figure S6. Activity of enzyme control hydrogels.
Figure S7. Impact of EV and liposome loaded hydrogels on inflammatory markers.

Supplementary Methods

EV isolation and enzyme loading: human mesenchymal stem cells (hMSCs) were maintained in TheraPEAK™ MSCGM-CD™ Mesenchymal Stem Cell Growth Medium (Lonza). TheraPEAK™ is a chemically defined serum-free medium that contains human albumin, recombinant human insulin, pasteurized human transferrin, HEPES, and L-glutamine. Cells were conditioned for 2-3 days in the medium; conditioned medium was continuously collected, centrifuged at 300 x *g* for 15 min and the supernatant stored at -80 °C until further use. For EV isolation and loading, our previously established protocol[1] was adapted. Briefly, 70-140 mL supernatants (corresponding to 4-16 x 10^6 hMSC cells) were centrifuged at 4000 x *g* for 15 min and pelleted at 120,000 x *g* for 2 h. Supernatants were removed and the EV pellet re-suspended in typically 400-600 µL PBS (**Figure S1a**). Subsequently, EVs were mixed with β-glucuronidase (Sigma G7646, final 1.5 mg/mL *w/v*) and saponin (Sigma 47036, final 1 mg/mL) and incubated at room temperature for 10 min. Then loaded EVs were purified by size exclusion chromatography (SEC) using sepharose CL-2B (17 mL, eluent PBS), where EVs typically eluted between 6 and 8 mL fraction volume. Pooled fractions were centrifuged using Amicon Ultra-15 centrifugal units (molecular weight cut-off 100 kDa) for 5 min at 3000 x *g* to render a final volume of 500-1000 µL. EV size and concentration was measured by nanoparticle tracking analysis (LM-10, Malvern Instruments, Figure S1a). Enzymatic activity was measured by mixing 125 µL of each fraction with 25 µL of fluorescein di-β-D-glucuronide (50 mM, Thermo) and incubation at 37 °C. Fluorescein release was measured at 0 and 16 h on an EnSpire microplate reader (PerkinElmer) at 495/520 nm (excitation/emission) and the average fluorescence signal produced by 10^8 EVs calculated (Figure S1c). Samples were stored at -80 °C until further use.

Liposome preparation and enzyme loading: Liposomes were prepared as described before.[1] Briefly, lipid films of 1,2-dimyristoyl-*sn*-glycero-3-phosphocholine/1,2-dipalmitoyl-*sn*-glycero-3-phosphocholine (2/3 mass ratio, Avanti) were hydrated with 1 mL of β-glucuronidase in PBS (1.5 mg/mL, *w*/*v*) at 37 °C. Suspensions were subsequently extruded 21x through a 200 nm membrane using a hand-held mini extruder with heating block (Avanti) (Figure S1b). Loaded liposomes were purified by SEC and characterized similar to the EVs (Figure S1c).

Preparation and characterization of hydrogels: 720 mg of poly(vinyl alcohol) (PVA, average Mw 89,000-98,000 g/mol, Sigma 341584) were dissolved in 6 mL of MilliQ water. The PVA solution was autoclaved without the lid (~1 h, 115-121°C) and cooled down to room temperature. 150 µL of 20 mg/mL Poly–L–lysine (average Mw 30,000-70,000 g/mol, Sigma P2636) were added and mixed gently into the PVA solution to ensure a homogeneous distribution. Hydrogels were fabricated using silicone rubber templates attached to the matt, hydrophobic side of SuperFrost Plus glass slides. In each template 90 µL PVA solution were mixed with 50 µL of EVs or Liposomes (50 or 100 × 10^8 vesicles/mL), free glucuronidase (0.1 mg/mL) or PBS and crosslinked with 2 x 25 µL of poly(ethylene glycol) (PEG, average Mw 400 g/mol, Sigma 202398). Air bubbles were removed using an 18G needle and gels were left to crosslink for 3 h in a petri dish with wet paper tissue to maintain humidity. Gels were further stabilized by immersion in PEG solution in 6 well plates overnight and subsequently washed three times with PBS. The number of vesicles per gel was calculated based on the geometrical volume of each gel and the concentration of EVs or liposomes added. It was estimated to be 1.7 and 3.5 × 10^8 vesicles/gel for 50 and 100 × 10^8 vesicles/mL concentration used, respectively.

Hydrogels were mechanically tested in unconfined compression using an ElectroForce (Bose) equipped with a 22.5 N load cell. For all tests, sample dimensions were measured in

wet state using digital calipers. Samples were pre-loaded to 0.05 N and compressed to 10% strain at a crosshead speed of 0.5% strain/min. The compressive modulus was calculated from the linear region of the stress-strain curve. Each experiment was repeated with $n = 5$.

For stability assessments, gels were incubated in PBS at 24 °C or 37 °C and their weight was monitored over 15 days. Each sample was normalized to the initial weight (**Figure S2a**).

Enzyme release was assessed by incubating the hydrogels in fresh PBS at 37 °C. After 48 h and 7 days 150 µL of supernatant was collected and centrifuged at 10,000 x *g* for 10 min. 125 µL of supernatant was mixed with 25 µL of fluorescein di-β-D-glucuronide (50 mM) at 37 °C. Fluorescein release was measured at 0 and 16 h on an EnSpire microplate reader (PerkinElmer) at 495/520 nm (excitation/emission) and the average fluorescence signal normalized to the 0 h value (Figure S2b). Each experiment was repeated with $n = 5$-7.

Fluorescence microscopy: EVs were labeled with a green fluorescent membrane dye PKH67 using the supplier's kit (Sigma). Typically, 200 µL of EVs were diluted in 400 µL diluent C and added to a mixture of 4 µL PKH67 stock in 196 µL diluent C. After incubation for 10 min at room temperature, EVs were purified by SEC, characterized, and loaded into hydrogels as described above. Hydrogels containing PKH67-labeled EVs were imaged using an inverted confocal laser scanning microscope (Leica TCS SP5) with incubation chamber to maintain gel hydration during imaging. In general, an APO 63x/1.4NA oil immersion objective was used for bright field imaging of gels and fluorescence detection in the green channel with a 488 nm (green) excitation laser.

Assessment of TNF-α gene and protein expression: Bone marrow cells were harvested from 6-8 week old CD-1 mice (Charles River) and matured to macrophages over 7 days in culture medium containing macrophage colony stimulating factor according to a well-established

protocol.[2] Bone marrow derived macrophages (BMDMs) were incubated with EV-hydrogels and liposome-hydrogels loaded with 3.5×10^8 β-glucuronidase-encapsulated vesicles per gel. Bacterial lipopolysaccharide (100 ng/mL, from *Escherichia coli* 026:B6, Sigma L2654) and curcumin-β-D-glucuronide (0.8 µM) were added and were incubated for 24 h at 37 °C and 5% CO_2. RNA was isolated from macrophages using TRIzol (Thermofisher) and converted to cDNA using a High-Capacity cDNA Reverse Transcription kit (Qiagen). TNF gene expression was measured via qPCR using TaqMan Gene Expression Assays (Thermofisher) according the manufacturer instructions, and normalized to GAPDH. TNF alpha protein concentration in cell culture supernatant was analyzed using Mouse TNFα Quantikine ELISA kit (R&D Systems) as per supplier's instructions. Obtained gene and protein expression values were normalized to LPS containing control samples. Experiments were completed on 3 independent cell isolations.

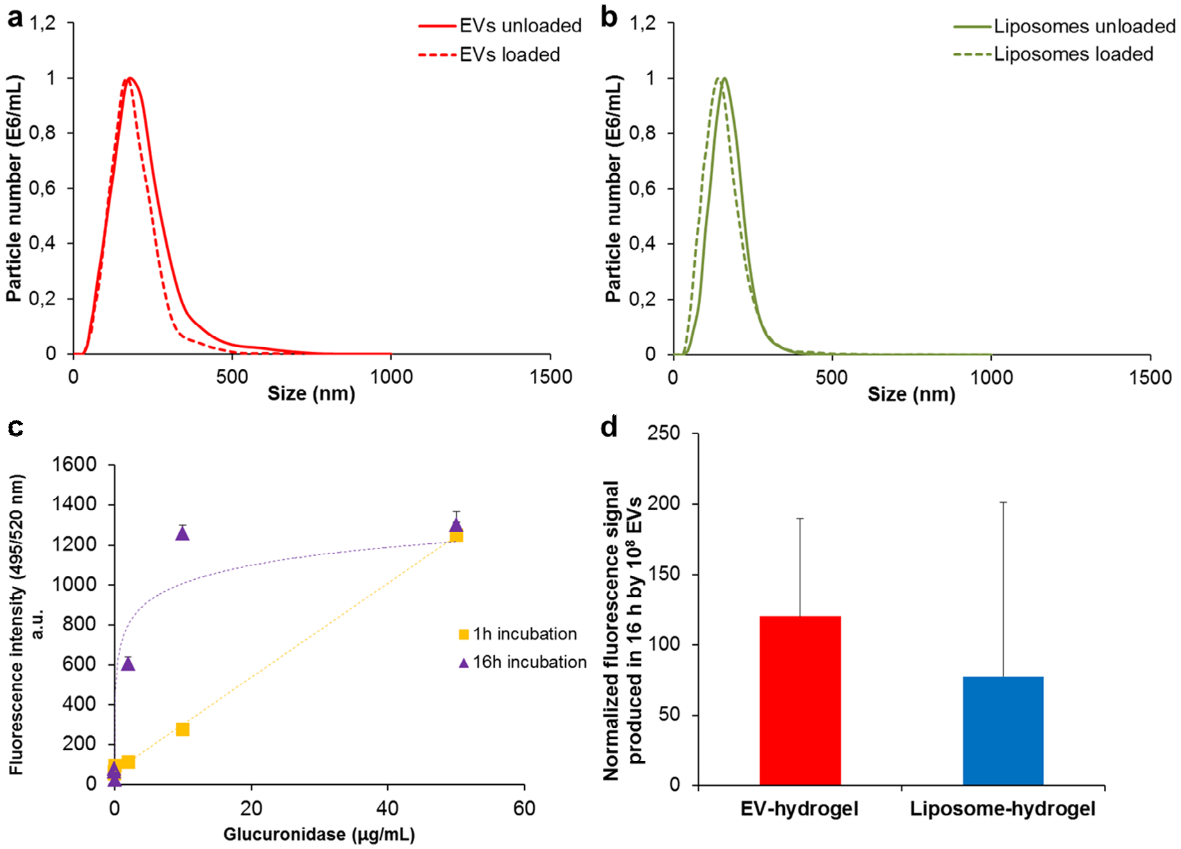

Figure S1. Characterization and loading of EVs and liposomes. a) Representative size distribution of EVs from mesenchymal stem cells before and after encapsulation of β-glucuronidase. b) Representative size distribution of liposomes before and after encapsulation of β-glucuronidase. c) Fluorescence intensity measured at 495 nm excitation and 520 nm emission wavelengths for incubation of glucuronidase in PBS with fluorescein di-β-D-glucuronide (8.3 mM) and at different timepoints (1 h and 16 h). d) Encapsulation efficiency of β-glucuronidase into EVs and liposomes was determined through monitoring the conversion of fluorescein di-β-D-glucuronide to fluorescein. Values are represented as mean ± SD, *n* = 9-12.

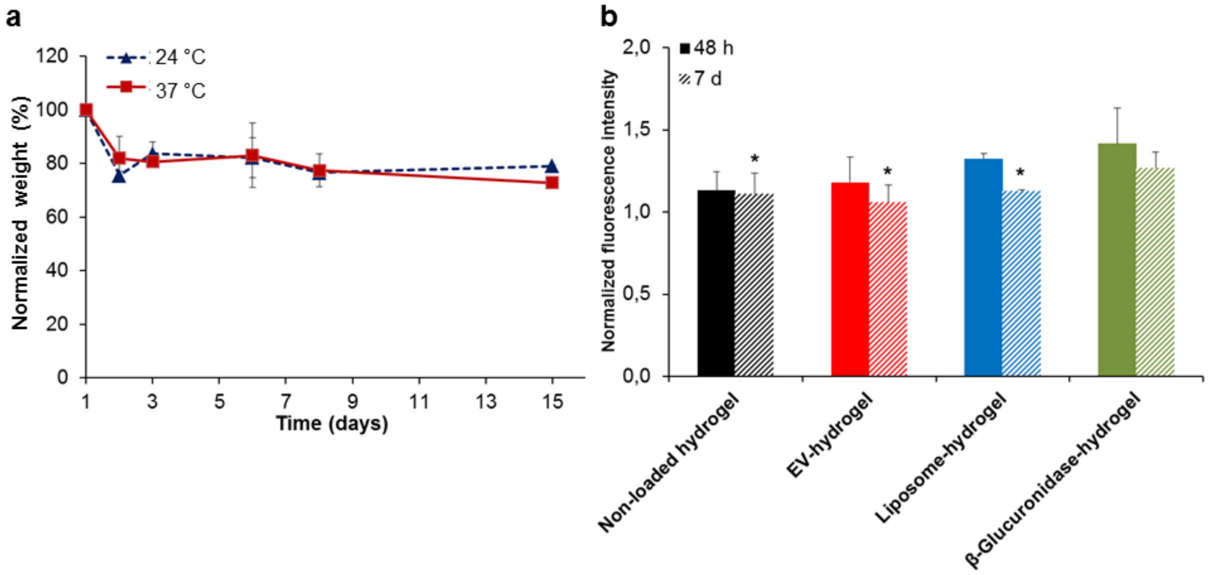

Figure S2. Stability of hydrogels upon storage. a) Weight loss of EV-hydrogels at 24 °C and 37 °C during 15 days of storage in PBS. b) Measurement of enzyme activity of supernatants of hydrogels at 37 °C incubated for 48 h or 7 days. Hydrogels contained no vesicles (control gel), or contained EV- and liposome-encapsulated glucuronidase, or free enzyme. Values are represented as mean ± SD, n = 5-7, *p<0.05 *vs.* glucuronidase, ANOVA followed by Fisher *post-hoc* test.

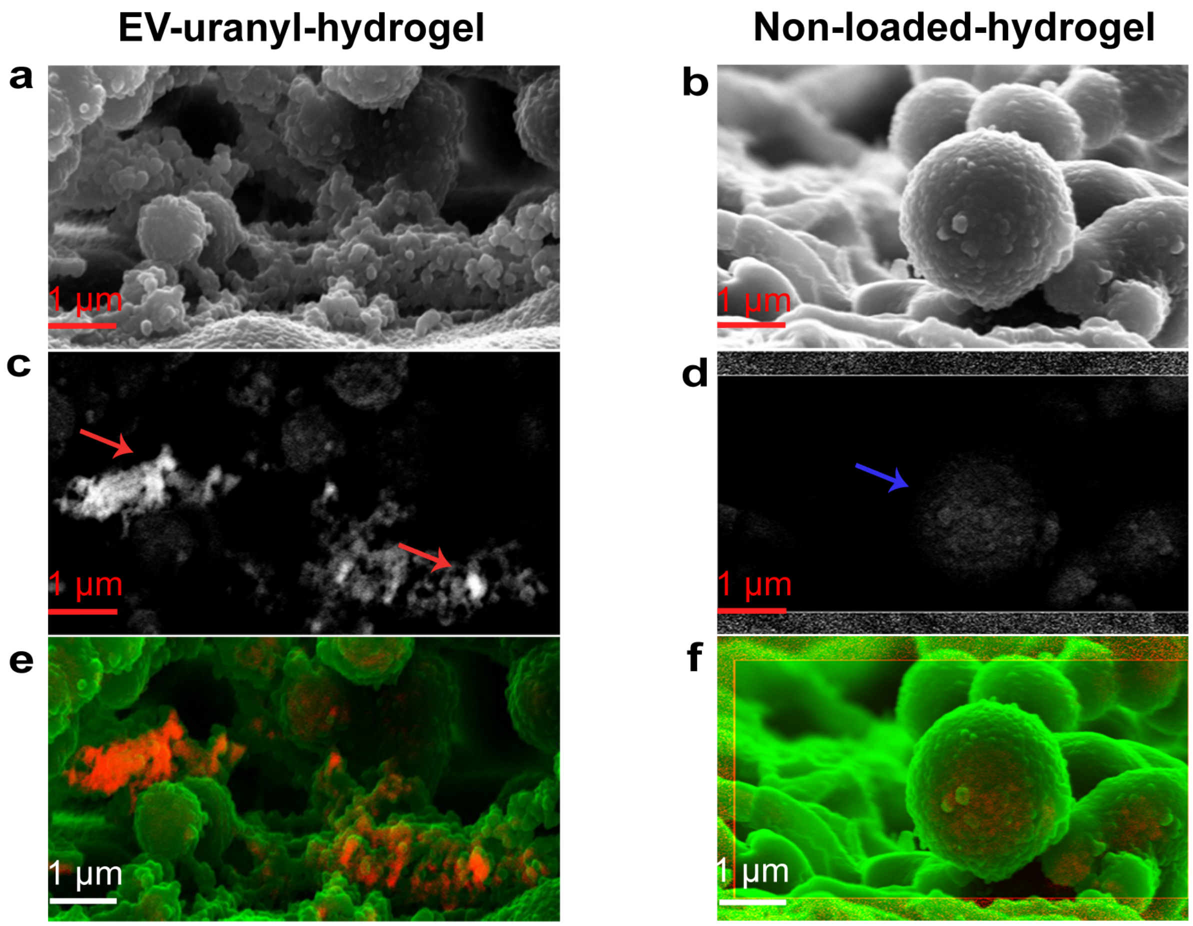

Figure S3. Supplementary density-dependent scanning electron microscopy imaging of EV-uranyl loaded hydrogel and non-loaded hydrogel. Empty poly(vinyl alcohol) hydrogels or hydrogels containing EVs were imaged by DDC-SEM. Imaging of hydrogels revealed optically more dense uranyl-labeled EVs (EV-uranyl, 3.5×10^8 EVs/gel, indicated by arrows) which were not observed in control gels (non-loaded-hydrogel). Images were obtained by in-lens electron detector (a and b) and in backscattered electron mode (c and d). They were recorded at a tilt angle of 70° and post-corrected to a tilt angle of 0° for display. The density-dependent color SEM analysis was executed by assigning the in-lens or secondary electron image to the green channel and the backscattering signal to the red channel (e and f).

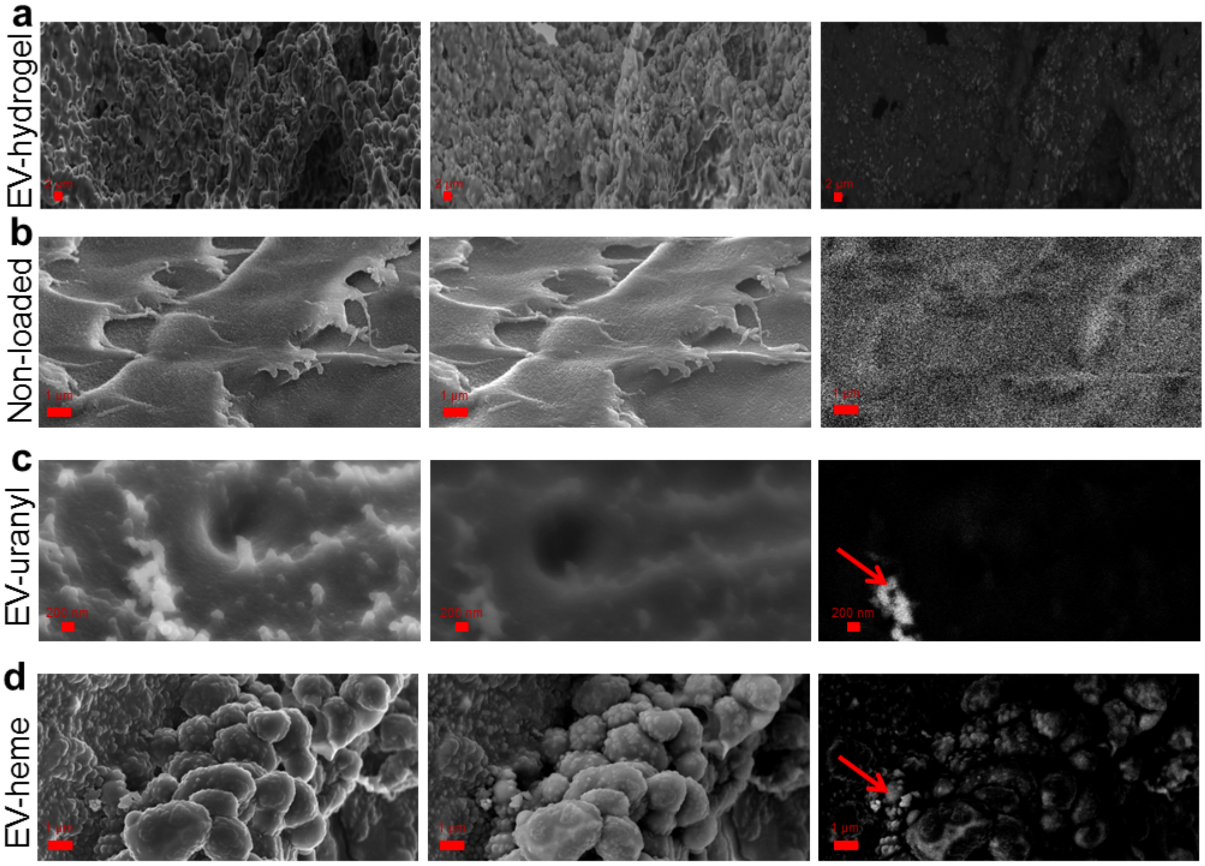

Figure S4. Complementary scanning electron micrographs of a) gels with unlabeled EVs or b) non-loaded gels (control gel). c) and d) PVA hydrogels containing optically more dense uranyl-labelled EVs (EV-uranyl) or heme-labelled EVs (EV-heme) as indicated by arrows. Representative images were obtained by in-lens electron detector (left column) and conventional secondary electron detector (middle column), and in backscattered electron mode (right column).

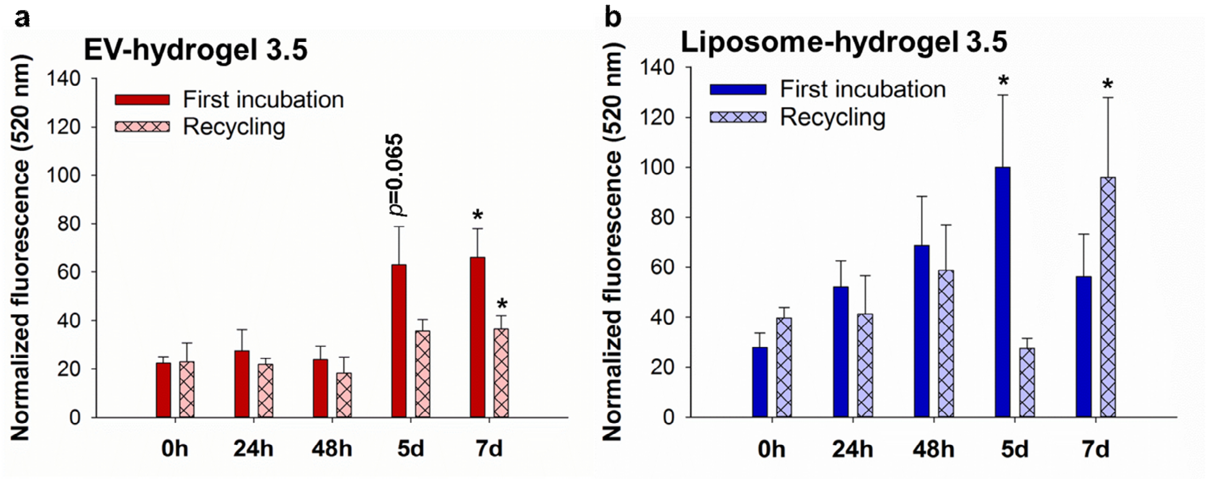

Figure S5. Activity of EV and liposome loaded hydrogels. Enzymatic activity of vesicle-loaded hydrogels. EV-hydrogels and liposome-hydrogels were loaded with 3.5×10^8 β-glucuronidase-encapsulated vesicles per gel. Incubation of a) EV-encapsulated β-glucuronidase in hydrogels or b) liposome-encapsulated β-glucuronidase in hydrogels with fluorescein di-β-D-glucuronide for up to 7 days (first incubation). Enzymatic cleavage was assessed by measuring increasing cumulative fluorescence produced by fluorescein. After 7 days, gels were washed thoroughly with PBS and incubated with fresh fluorescein di-β-D-glucuronide substrate to assess the enzyme activity upon long-term application (recycling). Values are represented as mean ± SD, n = 3-5, *p<0.05 vs hydrogels at 0 h (ANOVA on Ranks with Dunn's *post-hoc* test was performed on raw data). Normalization was executed against PBS control sample (set to 0%, not included) and the highest observed fluorescein release (100%). In a and b, 0% and 100% are equal to facilitate comparison between both cycles.

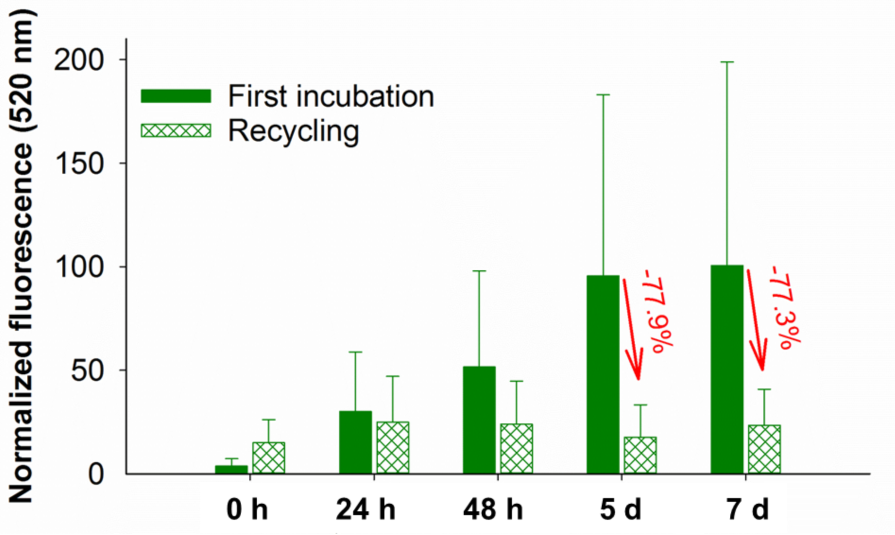

Figure S6. Activity of non-encapsulated β-glucuronidase in hydrogels over time. Incubation of hydrogels containing free β-glucuronidase (0.1 mg/mL) with fluorescein di-β-D-glucuronide for up to 7 days. Enzymatic cleavage was assessed by measuring increasing fluorescence produced by fluorescein. After 7 days, gels were washed thoroughly with PBS and incubated with fresh fluorescein di-β-D-glucuronide substrate to assess the enzyme activity upon long-term application. Values are represented as mean ± SD, *n* = 3-5, normalization was executed against PBS control sample (set to 0%, not included) and the highest observed fluorescein release (100%). Arrows indicate percentage loss of activity after 5 and 7 days of repeated incubation.

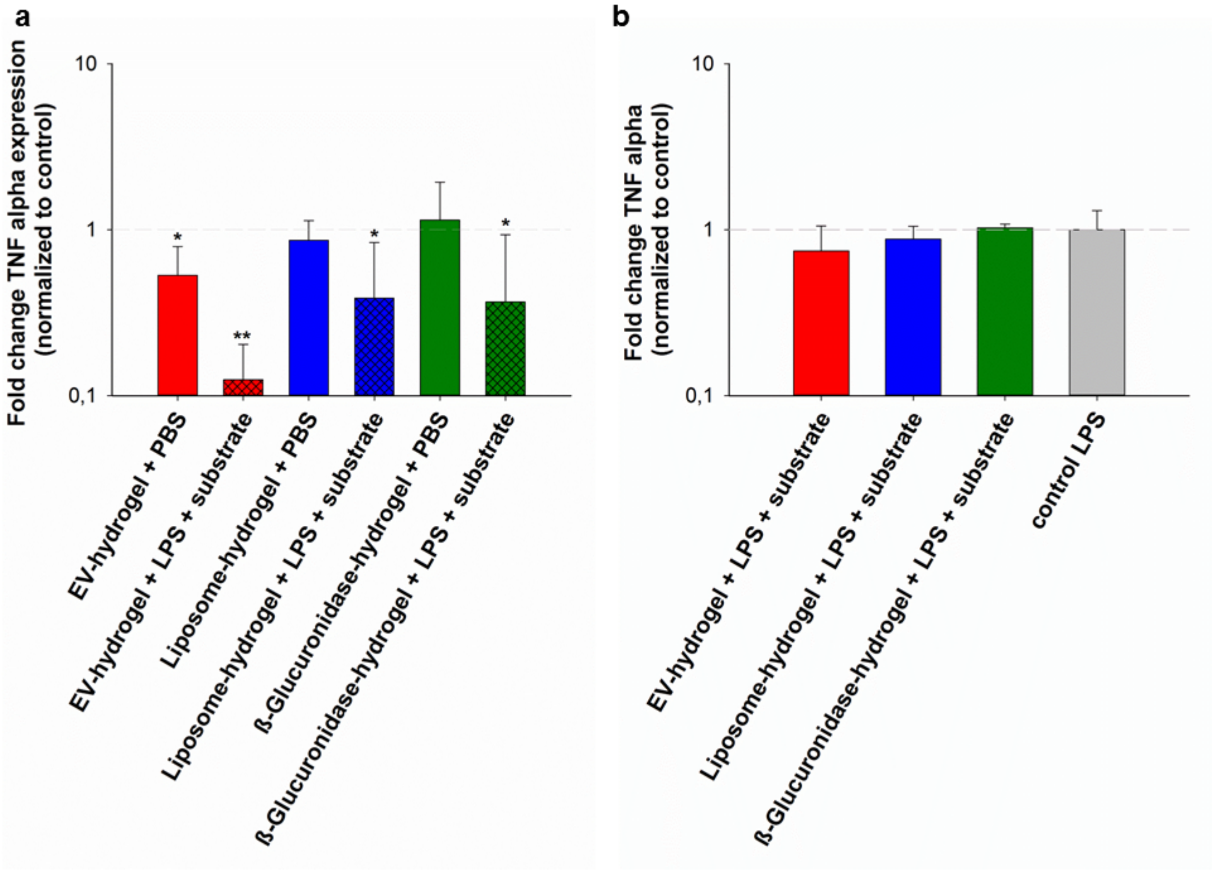

Figure S7. Impact of EV and liposome loaded hydrogels on inflammatory markers. EV-hydrogels and liposome-hydrogels were loaded with 3.5 × 10^8 β-glucuronidase-encapsulated vesicles per gel. Gels were incubated with primary bone-marrow derived murine macrophages and with LPS plus 0.8 µM curcumin-β-D-glucuronide, or PBS alone for 48 h. Gene expression and protein concentration of TNF alpha was assessed by PCR and ELISA. Values are represented as mean ± SD, $n = 3$, *$p<0.05$ vs β-glucuronidase-hydrogel + PBS (in a) (one-way ANOVA with Fisher *post-hoc* test). Normalization was executed against a control sample containing only LPS in cell culture medium.

Supplementary references

[1] G. Fuhrmann, A. Serio, M. Mazo, R. Nair, M. M. Stevens, J. Control. Release 2015, 205, 35.

[2] X. Zhang, R. Goncalves, D. M. Mosser, in *Current Protocols in Immunology*, John Wiley & Sons, Inc., 2001.
